# Carbonic Anhydrases in Cnidarians: Novel Perspectives from the Octocorallian *Corallium rubrum*

**DOI:** 10.1371/journal.pone.0160368

**Published:** 2016-08-11

**Authors:** Carine Le Goff, Philippe Ganot, Didier Zoccola, Natacha Caminiti-Segonds, Denis Allemand, Sylvie Tambutté

**Affiliations:** Centre Scientifique de Monaco, Monaco, Monaco; King Abdullah University of Science and Technology, SAUDI ARABIA

## Abstract

Although the ability to elaborate calcium carbonate biominerals was apparently gained independently during animal evolution, members of the alpha carbonic anhydrases (α-CAs) family, which catalyze the interconversion of CO_2_ into HCO_3_^-^, are involved in the biomineralization process across metazoans. In the Mediterranean red coral *Corallium rubrum*, inhibition studies suggest an essential role of CAs in the synthesis of two biominerals produced in this octocoral, the axial skeleton and the sclerites. Hitherto no molecular characterization of these enzymes was available. In the present study we determined the complete set of α-CAs in *C*. *rubrum* by data mining the genome and transcriptome, and measured their differential gene expression between calcifying and non-calcifying tissues. We identified six isozymes (CruCA1-6), one cytosolic and five secreted/membrane-bound among which one lacked two of the three zinc-binding histidines and was so referred to as a carbonic anhydrase related protein (CARP). One secreted isozyme (CruCA4) showed specific expression both by qPCR and western-blot in the calcifying tissues, suggesting its involvement in biomineralization. Moreover, phylogenetic analyses of α-CAs, identified in six representative cnidarians with complete genome, support an independent recruitment of α-CAs for biomineralization within anthozoans. Finally, characterization of cnidarian CARPs highlighted two families: the monophyletic cytosolic CARPs, and the polyphyletic secreted CARPs harboring a cnidarian specific cysteine disulfide bridge. Alignment of the cytosolic CARPs revealed an evolutionary conserved R-H-Q motif in place of the characteristic zinc-binding H-H-H necessary for the catalytic function of α-CAs.

## Introduction

Carbonic anhydrases (CAs) form a superfamily of mostly zinc-binding metalloenzymes that catalyze, by approximately one million fold [1], the interconversion of carbon dioxide and bicarbonate: ${\text{CO}}_{2}+{\text{H}}_{2}\text{O }↔{\text{HCO}}_{3}^{-}\;+{\text{H}}^{+}$. These enzymes are a critical component of the acid-base balance and play roles in numerous biological functions [2]. CAs are found in the three domains of life (Eubacteria, Archaea and Eukaryota) and are encoded by six distinct and evolutionarily unrelated gene families: α-CAs, β-CAs, γ-CAs, δ-CAs, ζ-CAs [3,4] and η-CAs [5]. In metazoans, CAs belong predominantly to the α-CA family, though β-CA family members have been recently identified [6–8]. The α-CA family, which is the best studied CA family, comprises numerous isoforms that differ by their enzymatic activity, kinetic properties, sensitivity to inhibitors, and their tissular and subcellular distribution. They are usually classified according to their subcellular localization: cytosolic, mitochondrial, membrane-bound, or secreted. The typical structure of α-CA [9,10] is characterized by 1) three histidine residues (His94, His96, His119, human CAII numbering) that bind the zinc cation cofactor, 2) a proton shuttling residue (His64, human CAII numbering), placed at the entrance of the active site and responsible for converting zinc-bound water molecule to hydroxide ion, and 3) the gatekeeper residues (Glu106 and Thr199, human CAII numbering) which allow the optimal orientation of the zinc-bound hydroxide ion to enhance the nucleophilic attack of the substrate (CO_2_). Among the α-CAs identified in human, three are non-catalytic in regard to the reversible hydration of CO_2_ to HCO_3_^-^. These CARPs (Carbonic Anhydrase Related Proteins) correspond to the cytosolic CAVIII [11], and the secreted CAX [12] and XI [13]. Although their overall structure is highly homologous to the other human α-CAs, they lack at least one of the three zinc-binding histidine residues, causing catalytic inactivity [3,14–16]. In spite of being classified as “acatalytic”, CARPs have been identified in most taxa of the metazoan lineage [17]. They are highly conserved across vertebrates, and evolved either from a common ancestor in bilaterians [18], or have been invented at least twice during metazoan evolution [19], implying positive selection. However the precise biological role of CARPs within or outside the cell remains unknown.

Recently, about thirty metazoan calcifying species where α-CAs are present and potentially involved in the deposition of calcium carbonate biominerals were listed [20]. The involvement of α-CAs in the formation of carbonate skeletons was reported in Porifera, the oldest metazoan phylum with the ability to produce biominerals [19,21]. Whereas it is accepted that these enzymes are part of a “biomineralization/skeletogenesis toolkit” upon which metazoans have elaborated their biomineralization strategies, the exact role of α-CAs in this process is still yet not well understood [19,21,22]. In corals, some information on the potential role of α-CAs in biomineralization comes from their localization in the tissues: in the scleractinian coral *Stylophora pistillata*, a secreted α-CA has been identified in the calcifying cells, which are responsible for skeleton formation [23]. This CA may help catalyzing the supply of inorganic carbon in the calcifying medium. As in other metazoans’ calcium carbonate biominerals, such as calcareous sponges spicules [19], demosponges basal skeletons [21], molluscan shells [24,25] and statocyts [26], sea urchin tests and spines [25,27], CAs have also been found in the organic matrices of coral skeletons [28–33]. These CAs could play a key role in the precipitation of calcium carbonate.

Cnidarians are principally divided in two major groups: anthozoans, comprising hexacorallians and octocorallians, and medusozoans. To date, most studies on cnidarian α-CAs have mainly concentrated on hexacorallians (for reviews see [34]). In octocorallians, CA activity was only reported in the organic matrices of the endoskeletal sclerites of *Lobophytum crassum* [35,36] and skeleton of the red coral *Corallium rubrum* [25]. In order to provide informative knowledge on α-CAs in octocorals, we focused our study on the Mediterranean red coral *C*. *rubrum*. This species, which is famous in jewelry for its red colored skeleton, elaborates two biominerals: the axial skeleton and the sclerites. Both are composed of a mineral fraction made of calcium carbonate (CaCO_3_) crystallized in the form of high-magnesium calcite [37] and an organic fraction [38]. However, they differ in their shape, size and their protein content [39]. The axial skeleton growth results from an extracellular process, *i*.*e*. deposition of CaCO_3_ by the skeletogenic epithelium [40,41]. Octocorals sclerites are supposedly initially formed in intracellular vesicles or vacuoles within primary scleroblasts present throughout the mesoglea of the coenenchyme (tissue connecting the polyps), and then released outside the cells where it is yet unclear whether they continue growing [42–48]. Nevertheless, previous pharmacological studies have shown that carbonic anhydrases are involved in the formation of these two biominerals since Diamox, a specific CAs inhibitor, inhibits the deposition of calcium in both the axial skeleton and the sclerites (72 and 52% respectively) of *C*. *rubrum* [49].

We here addressed the molecular identification of all α-CAs present in the Mediterranean red coral *C*. *rubrum* and pointed to the α-CA isozyme(s) potentially involved in its calcification process. We next added to our analyses the up-to-date set of α-CAs encoded in cnidarian organisms with complete genome, predicted their cellular localization, and considered their evolutionary history within Cnidaria. Furthermore, we focused on the cnidarian CARPs to investigate the structural evolution of their “acatalytic site” across anthozoans.

## Material and Methods

### Bioinformatic analyses

#### Sequences

The *C*. *rubrum* CAs, named CruCAs, were identified from unpublished transcriptome and genome assemblies of *C*. *rubrum* using human (15 HsCAs) and *Stylophora pistillata* (16 SpiCAs) CA proteins as initial query sequences. The working assembly transcriptome of *C*. *rubrum* is accessible on the Centre Scientifique de Monaco blast server: http://data.centrescientifique.mc/blast/blast.php. To generate *C*. *rubrum* transcriptome, RNA from three different colonies was extracted using standard Trizol extraction (see below). Strand specific paired-end libraries were constructed and sequenced in multiplex on a one lane Illumina HiSeq 2000 sequencer (GeneCoreFacility, EMBL, Germany). Reads (4.8 10^8^ x 101 bp) were assembled using the Trinity pipeline (r2014-07-17) with default parameter. Approx. 34000 protein coding sequences were generated. Nucleotide sequences specific for CruCAs are provided in S1 Table. The initial set of SpiCA isoforms, comprising STPCA [23] (renamed SpiCA1) and STPCA2 [50] (SpiCA2), was completed using *S*. *pistillata* ESTs obtained after Illumina sequencing [51]. SpiCA1 was extended and updated. Sequences from *Acropora millepora* were retrieved from Compagen (www.compagen.org), NCBI EST and TSA databases; sequences from *Nematostella vectensis* were retrieved from the JGI genomic database (http://genome.jgi-psf.org/) and from the Vienna database (https://figshare.com/articles/Nematostella_vectensis_transcriptome_and_gene_models_v2_0/807696); sequences from *Aiptasia pallida* were retrieved from Pringle Lab ESTs database (http://pringlelab.stanford.edu) [52]; and sequences from *Hydra magnipapillata* were retrieved from GenBank. Sequences from the two calcareous sponges, *Sycon ciliatum* and *Leucosolenia complicata*, were retrieved from UniProt knowledgebase. Some of these databases are available on the Centre Scientifique de Monaco blast server: http://data.centrescientifique.mc/blast/blast.php. The CARP sequences outside those species were obtained from the anthozoan subdivision of the NCBI EST and TSA databases. The newly identified sequences reported in this paper (*C*. *rubrum* [KU557743 to KU557748], *S*. *pistillata* [KU557749 to KU557763] *and A*. *pallida* [KU557764 to KU557773]) have been deposited in the GenBank database and nucleotide sequences are reported in S1 Table.

#### *In silico* analyses

Data mining for cnidarian CAs were performed using Blast program suite. Homology was assessed by reverse Blast against NCBI non-redundant database and domain analysis with Pfam. Molecular weight and isoelectric point were computed with Compute PI/MW on the ExPASy server (web.expasy.org/compute_pi/). Signal peptide, transmembrane domains, N-/O- glycosylations, and phosphorylation sites were predicted using SignalP v4.1, TMHMM v2.0, NetNGlyc v1.0, NetOGlyc v4.0, and NetPhos v2.0 respectively, using the Center for Biological Sequences Analysis (CBS) servers (www.cbs.dtu.dk/services/). GPI (glycosylphosphatidylinositol) anchoring site prediction was performed using the big-PI predictor (mendel.imp.ac.at/gpi/cgi-bin/gpi_pred.cgi) and PredGPI (http://gpcr.biocomp.unibo.it/predgpi/pred.htm). Alignment of amino acid sequences of CruCAs was performed using Clustal Omega (www.ebi.ac.uk/Tools/msa/clustalo/) and edited by GeneDoc software [53].

#### Phylogenetic analysis

Three different alignment methods (Clustal Omega, Muscle and MAFFT) were used. Best fitted model, calculated using ProtTest v3.4 [54], was WAG+G+F in every case. The three phylogenetic trees were constructed with the Maximum Likelihood method using PhyML [55] to compare the congruence of the resulting trees. Little variation was observed between PhyML trees (S2 Fig). For comparative purposes with other published cnidarian phylogeny [20,23,34] we show here the phylogeny inferred from the Clustal Omega alignment. Only the part corresponding to the CA catalytic domain was considered for further analysis. Bayesian inference was performed with MrBayes v3.2 using the following parameters (see S1 Appendix): lset nst = 6 rates = invgamma;\n prset applyto = (all) ratepr = variable;\n mcmcp ngen = 50000000 relburnin = yes burninfrac = 0.25 printfreq = 1000 samplefreq = 1000 nchains = 4 savebrlens = yes nruns = 2 mcmcdiagn = YES diagnfreq = 1000 checkfreq = 10000;\n scmc; sump; sumt; end.

#### Structural study

Predicted 3D structures of CruCAs and anthozoan CARPs were generated using the Swiss-Model protein structure homology-modelling server using best fitted CA structure models as templates and visualized with CCP4MG software (www.ccp4.ac.uk).

### Molecular and biochemical analyses

#### Biological material

Colonies of *C*. *rubrum* (Linnaeus, 1758) were collected at 10 meters depth near Marseille (Plane Island, Gulf of Lion: 43° 11.190'N, 5° 23.470'E) by IMBE/Marseille. Permission was delivered by the Maritime Prefect of the Bouches-du-Rhône, France. Note that when sampling a coral branch, healing occurs within two weeks excluding impact on the species and specimen. Colonies were transferred to Monaco and maintained in an open-circuit seawater aquarium supplied with filtered Mediterranean seawater (18°C ± 2°C). Colonies were fed with mixture of frozen artemia, rotifers and red plankton every working day.

#### Dissection

**Fig 1:**
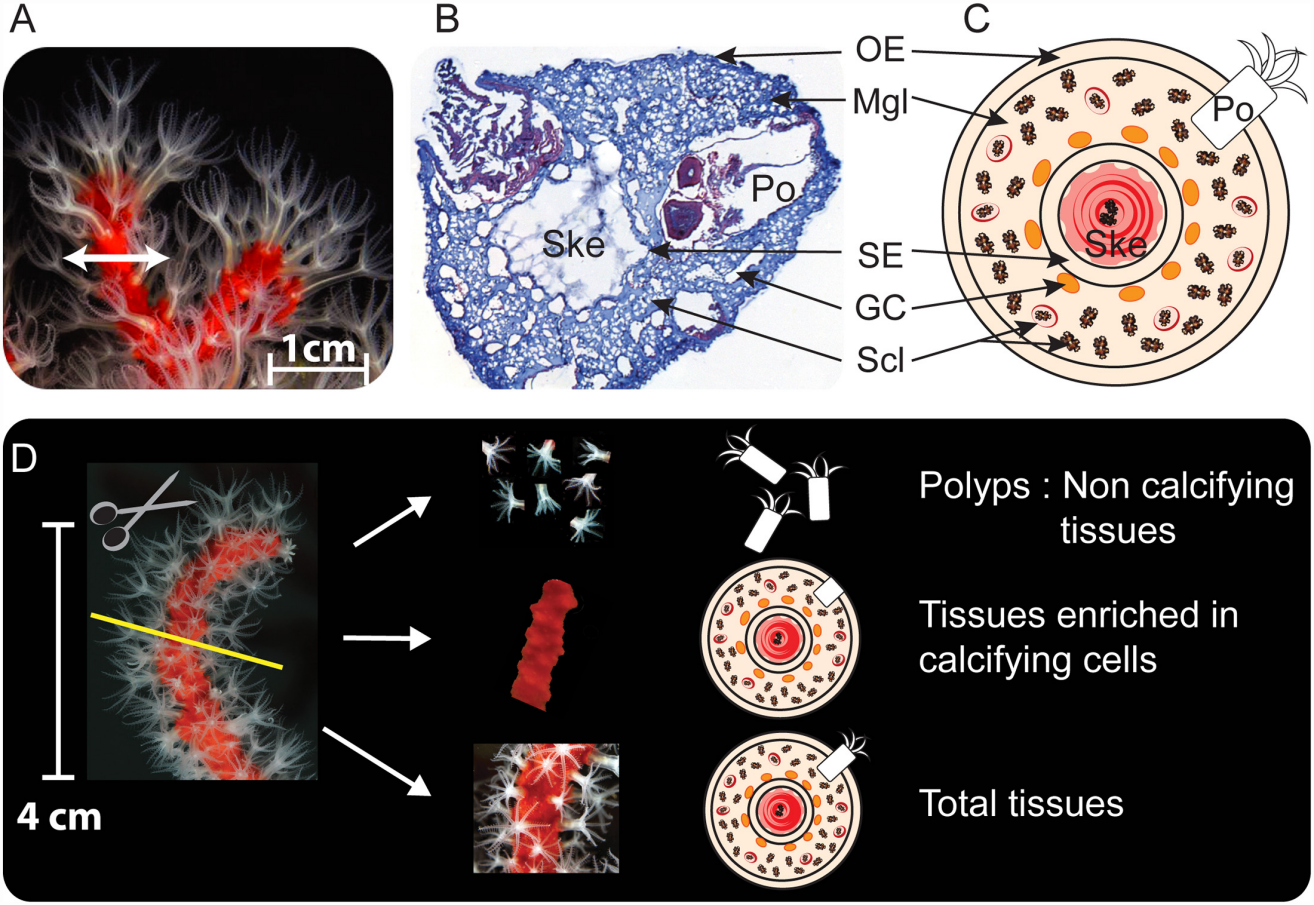
Histology of *C*. *rubrum* and dissection protocol to obtain the three different fractions. (A) Photo of a *C*. *rubrum* colony. (B) Transversal cryosection of a demineralized branch stained with Hemalin-Eosin and acetified Aniline blue. (C) Schematic representation of *C*. *rubrum* organization. OE: Oral epithelium; Mgl: Mesoglea; Po: Polyps; Ske: Axial skeleton; SE: Skeletogenic epithelium; GC: Gastrodermal canals; Scl: Scleroblasts/Free sclerites. (D) Dissection protocol to obtain the three fractions for qPCR and western blot.

Three colonies, or branches (approximately 5 cm long), of *C*. *rubrum* with polyps extended were anesthetized in seawater supplemented with Tricaine methanesulfate (MS-222_Sigma) at a final concentration of 0.04% [56]. The different fractions obtained by microdissection under binocular microscope are represented in Fig 1. Each branch was cut into two equal parts; one half was put into plastic bag (Whirl-Pak^®^, Nasco, USA) and dip in liquid nitrogen. This part corresponds to the total tissue fraction. On the other half, the apparent part of polyps were cut out with 5 mm blade microdissection scissors (Vannas). These freshly dissected polyps correspond to the non-calcifying fraction. Polyps were transferred in 1 mL of Trizol reagent before being flash frozen. Polyps were then homogenized by hand using a potter-Elvehjem grinder, and volume of Trizol was adjusted to 10 mL. The rest of the branch, mostly devoid of polyps, corresponds to fraction enriched in calcifying cells, and was flash-frozen into plastic bag. This fraction and the total tissue fraction were then cryo-crushed (Freezer/Mill 6770, Spex Sample Prep). The resulting powders were each dissolved in 10 mL of Trizol reagent. Samples in Trizol were vortexed and incubated at room temperature (RT) for 10 min.

#### RNA extraction-DNase treatment-Reverse transcription

Two centrifugations (5 min, 10,000 g, 4°C and 10 min, 10,000 g, 4°C) were performed to remove skeletal and cellular debris. 2 mL of chloroform was added to the last supernatant, vortexed, incubated 3 min at RT, and centrifuged (3 min, 5,000 g, 4°C) to separate the aqueous phase containing the RNA from the phenol-chloroform phase. The aqueous phase was subjected to a precipitation with 2.5 mL of HSS [1.2 M NaCl; 0.8 M sodium citrate] and 2.5 mL of isopropanol, vigorously shaken and incubated 10 min at RT. Centrifugation (15 min, 12,500 g, RT) permitted to pellet the RNA. This pellet was resuspended twice in 10 mL cold ethanol 70%, vortexed, centrifuged (4 min, 8,000 g, 4°C) and dried at RT before to be resuspended in 50 μL RNase-free water. DNase treatment of samples was performed using RNase-free DNase I (Roche) followed by a RNA precipitation with 3 M sodium acetate pH 5.2. Total RNA was purified with the RNeasy mini kit (Qiagen). Reverse transcription were performed on 2 μg of RNA using 150 ng random primers with the Superscript III kit (Invitrogen) according to the instructions of the supplier.

#### Real-time PCR experiments

Specific primers of each CruCA were designed using Primer3 to produce 90–120 bp amplicons. Primers efficiencies were determined using standard curve analysis using 10-fold dilution series of pooled cDNAs. For each biological replicate, real time PCR were performed in technical triplicate with cDNAs diluted at a final concentration of 2 ng/μL and using the Express SYBR^®^ greenER^™^ SuperMix with premixed ROX (Invitrogen) in ABI 7300 Real-Time PCR System (Applied Biosystems). Four potential housekeeping genes (RPLP0, RPL22, RPL40, β-actine) were tested in qPCR based on previous experiments on other corals [57–59]. As in scleractinian corals, RPLP0 presented the most constant expression level across samples in *C*. *rubrum* (RPLP0: 0.02%).

Two methods were used to measure individual CruCA expression: (1) in total tissues fraction, the expression was calculated relative to RPLP0 using the adjusted 2dCt method taking into account primers efficiency; (2) expressions in tissues enriched in calcifying cells and polyps fraction, normalized to RPLP0 and relative to total tissues fraction were calculated according to the Pfaffl’s equation [52]. Results are presented as mean ± SEM. Differences were evaluated by one-way analysis of variance (ANOVA) or Student’s t-test. Results were considered statistically significant when P<0.05.

#### Protein extractions

Total tissue, calcifying and non-calcifying fractions of *C*. *rubrum* were prepared using the same dissection protocol as above-mentioned (Fig 1), except that fractions were directly flash-frozen and then crushed with microbeads (Retsch MM400) in 1 mL extraction buffer [50 mM Tris, 100 mM NaCl, 5 mM EDTA, 1% Triton X100, 0.1% Protease Inhibitors Cocktail (Sigma) and 5 mM phenanthroline]. Four differential centrifugations (from 3,500 g to 7,500 g at 4°C) were used to eliminate skeletal and cellular debris.

Antibodies—Synthesis of custom made polyclonal antibodies produced in rabbit was performed by Eurogentec. Purified antibodies were raised against peptide CPVNKRQVKASFQ (amino-acids 250–288) for CruCA3 and peptide NYRPVQDLNGRPVT (amino-acids 261–275) for CruCA4. Both sequences correspond to the C-terminus of the proteins in the linear and highly accessible part of their 3D structures.

#### Western blotting

Protein samples were denatured in Laemmli buffer (5 min at 95°C). Samples were run into 12% or 18% Criterion^™^ TGX^™^ precast gels (Bio-Rad). Proteins were transferred on polyvinylidene fluoride membrane (Trans-Blot^®^ Turbo^™^ Midi PVDF Transfer Packs_Bio-Rad). Membranes were blocked with 5% non-fat dry milk in TBS-Tween (TBS-T) (1 hour) and then incubated with primary antibody (CruCA3, 1:50; CruCA4, 1:125) in TBS-T solution containing 1% non-fat milk at 4°C overnight. After three rinses in TBS-T, membranes were incubated with anti-rabbit coupled to peroxidase (1:2,500; Sigma) for 1 hour in 1% non-fat milk-TBS-T solution, followed by six additional TBS-T rinses. The immunoreactive proteins were detected by enhanced chemiluminescence (Amersham ECL_GE Healthcare) and observed with FUSION FX7 (7026.WL/26MX, Vilber Lourmat). Membranes were then stripped in Restore^™^ Western Blot Stripping Buffer (Thermo Fisher) and probed with rabbit anti-actin (1:10,000; A-2668; Sigma) to ensure equal protein loading following the same protocol.

## Results

### Identification and characterization of CruCAs

Data mining of the genome and transcriptome of *C*. *rubrum* allowed us to identify six α-CA isoforms named CruCA1 to CruCA6. The presence of N-terminal signal peptides indicates that five of the six CruCAs (CruCA1-2-4-5-6) are secreted isozymes. CruCA5 is characterized by the presence of an additional C-terminal GPI-anchor site, which suggests that this isozyme is attached to the outer leaflet of the cellular membrane [60]. Since no signal peptide, no GPI-anchor site or no transmembrane domains were identified in CruCA3, this isozyme is likely a cytosolic CA. Table 1 presents the predicted open reading frame (ORF) length, the protein length, the molecular weight of each CruCA, their isoelectric point and number of glysosylation/phosphorylation sites. The range length of CruCAs is between 262 and 356 amino acids with an average molecular weight between 29 and 41 kDa. The isoelectric point (pI) of the majority of CruCAs is between 8 and 9.6 with the exception of CruCA3 which has an acidic pI of 5.88. We have also noted that the linear structure between the plasma membrane and the globular part of CruCA5 is highly glycosylated and phosphorylated.

**Table 1:** Characteristics of CruCA isozymes in *C*. *rubrum*.

					Number of predicted sites		
Protein	ORF length (bp)	Protein length (aa)	Molecular weight (kDa)	pI	N-glycosylation	O-glycosylation	Phosphorylation
CruCA1	1032	356	40.89	8.47	5	3	22
CruCA2	969	322	36.90	9.63	2	4	14
CruCA3	789	262	29.04	5.88	-	-	18
CruCA4	855	284	32.86	9.42	1	1	15
CruCA5	1008	335	36.06	8.77	5	20	27
CruCA6	846	281	32.06	8.26	0	3	14

Open Reading Frame (ORF) nucleotide length, amino acids (aa) protein length, calculated molecular weight, isoelectric point (pI), and number of predicted N- and O- glycosylation/phosphorylation sites is shown for each CruCA.

### Sequences alignment of CruCA proteins

**Fig 2:**
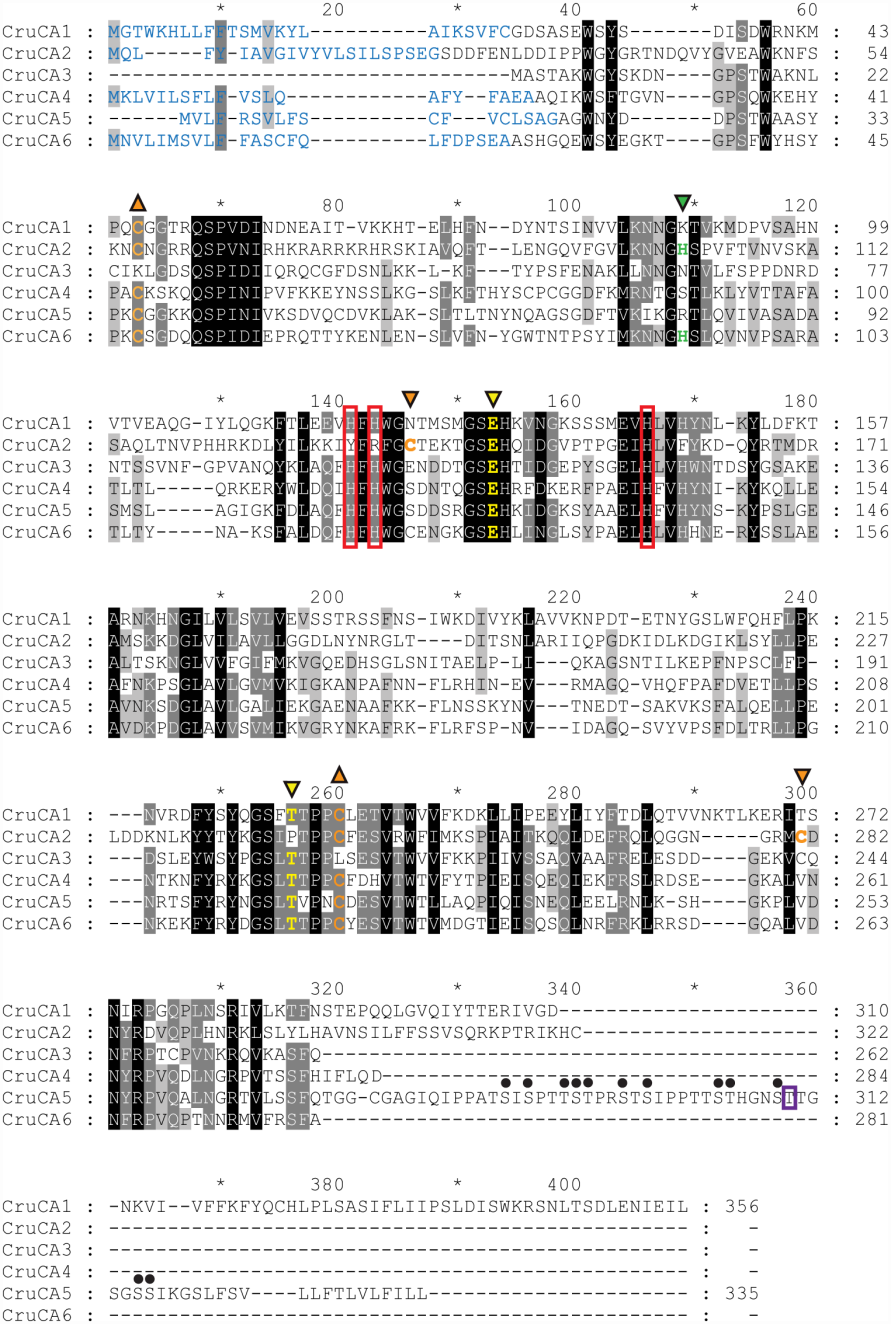
Clustal Omega alignment of CruCA proteins. 100%, 80%, and 60% conserved amino acids are shaded in black, dark grey, and light grey, respectively (GeneDoc software, score table BLOSUM62). The N-terminus signal peptide sequences are colored in blue. The predicted GPI-anchor site is indicated by a violet rectangle in the C-terminus of CruCA5. The three zinc-binding histidines are indicated by red boxes. The proton shuttle residue is indicated by inverted green triangle. The two gatekeeper residues are indicated by inverted yellow triangles. The cysteines involved in disulfide bonds are represented as follow: the Cys57~Cys250 (CruCA2 nomenclature), common to all extracellular cnidarian α-CAs, is shown with orange triangles; the Cys138~Cys284, specific to the secreted cnidarian α-CAs cluster, is shown with inverted orange triangles. The multiple phosphorylation sites in the C-terminus tail of CruCA5 are shown by a full circle above the predicted residues.

Alignment of CruCAs (Fig 2) shows that five of the six CruCAs (1, 3–6) have the three zinc-binding histidine residues. However, CruCA2 appears to lack CA activity because of the substitution of two functionally important histidines by a tyrosine and an arginine at position 133 and 135, respectively. This isozyme is therefore considered as a carbonic anhydrase related protein (CARP). The proton shuttle residue is conserved only in CruCA2 and CruCA6 (positions 101 and 92, respectively), whereas this histidine residue is substituted in the others CruCAs (CruCA1: Lys; CruCA3: Asn; CruCA4: Ser; CruCA5: Arg). The two gatekeeper residues are conserved in almost all CruCAs. CruCA2, the exception, has a proline residue (position 246) instead of the threonine, though a neighboring threonine is present. Of note is one highly conserved motif, QSPID/NI (positions 62–67), which seems not to be associated with α-CAs activity [18].

### Differential CruCAs gene expression

**Fig 3:**
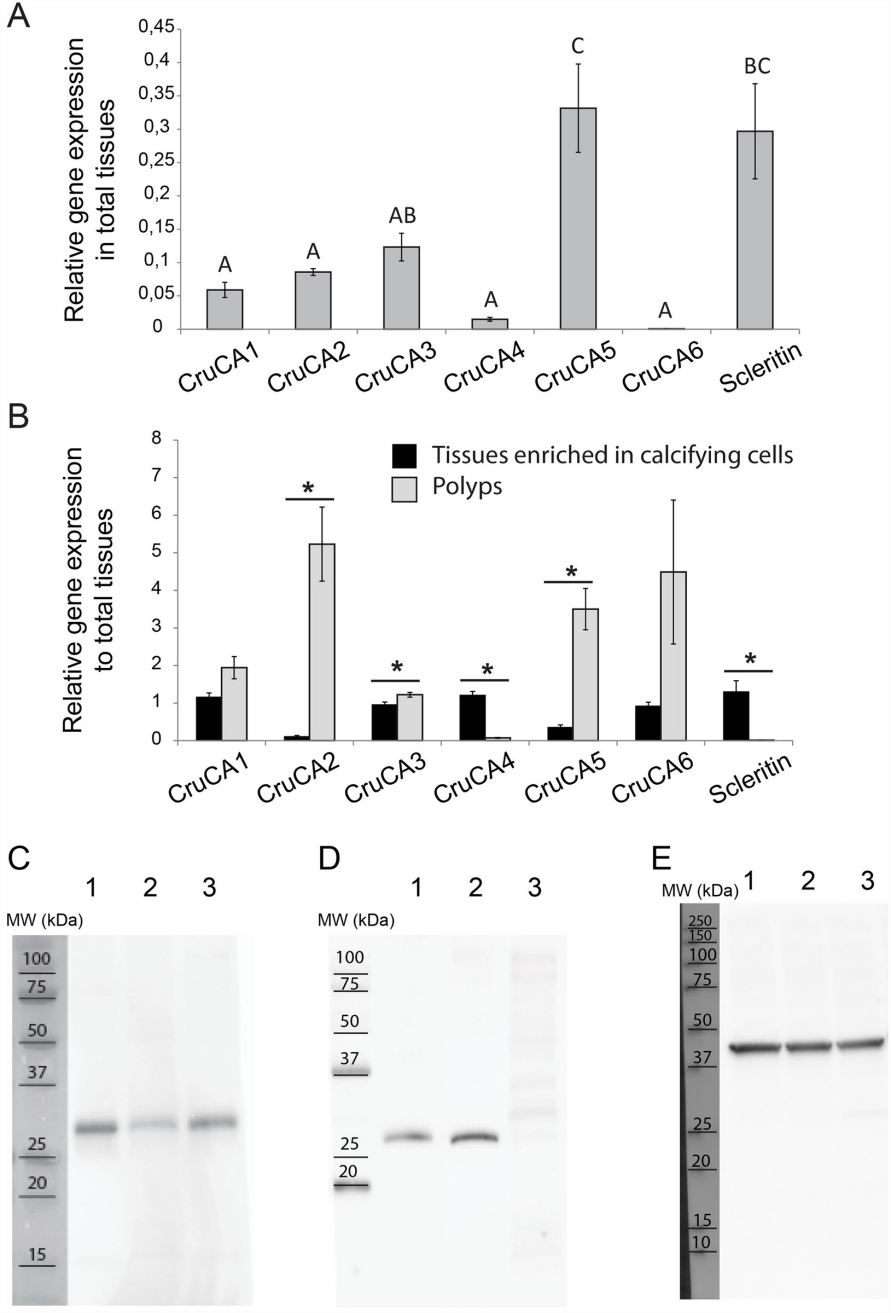
Relative gene expression of CruCA isozymes and western blot on proteins extracted from different tissue fractions of *C*. *rubrum*. (A) Gene expression in total tissues, relative to RPLP0 expression. Letters represent statistical differences based on one-way ANOVA (p<0.05). (B) Gene expression in tissues enriched in calcifying cells and in polyps, normalized to RPLP0 and relative to the expression in total tissues. Asterisks indicate values statistically different between fractions based on Student’s t-test with p<0.05. Error bars represent standard error of the mean. (C) Western blot with anti-CruCA3 (29 kDa) (12% polyacrylamide gel). (D) Western blot with anti-CruCA4 (33 kDa) (18% polyacrylamide gel). (E) Loading control western blot with anti-actin (42 kDa) (12% polyacrylamide gel). Lane 1: Total tissues extract; lane 2: Tissues enriched in calcifying cells fraction; lane 3: Polyps fraction. MW: molecular weight of Precision Plus Protein^™^ All Blue Standard (Bio-Rad).

In order to gain insight into the possible function of the different CruCAs, especially in regards to the biomineralization process, we measured by qPCR their relative gene expression levels in total tissue fraction as well as in two main compartments: the non-calcifying polyps and the tissues enriched in calcifying cells (scleroblasts and skeletogenic epithelium), corresponding to the coral tissues devoid of most polyps. As a positive control for our microdissection technique, we quantified the expression of the gene encoding the scleritin, a *C*. *rubrum* sclerite specific protein expressed by the scleroblasts [61]. Scleritin showed no expression in the non-calcifying fraction contrary to the calcifying and total tissue fractions. In the total tissue fraction (Fig 3*A*), CruCA5 was the most expressed isozyme. In contrast, CruCA4 and CruCA6 showed the lowest level of gene expression. When looking at the specific expression in calcifying and non-calcifying fractions (relative to total tissue fraction), all CruCAs except one showed predominant expression in the polyps’ fraction: CruCA4 was the only isozyme with a significant overexpression in tissues enriched in calcifying cells (Fig 3*B*).

In order to confirm the results obtained by qPCR on CruCA transcripts, we monitored the protein level of two CruCAs: CruCA3, the ubiquitous cytosolic α-CA, and CruCA4, the secreted and calcifying fraction specific α-CA. Custom-made purified antibodies against CruCA3 and CruCA4 were used in western blot experiments against total/calcifying/non-calcifying protein extracts of *C*. *rubrum*. We observed the same expression pattern as obtained by qPCR, with a higher level of expression in total tissue fractions and polyps for CruCA3 (Fig 3*C*), as opposed to CruCA4 that is mostly expressed in the fraction enriched in calcifying cells (Fig 3*D*).

### Phylogenetic and structural relationships of the cnidarian α-CAs

**Fig 4:**
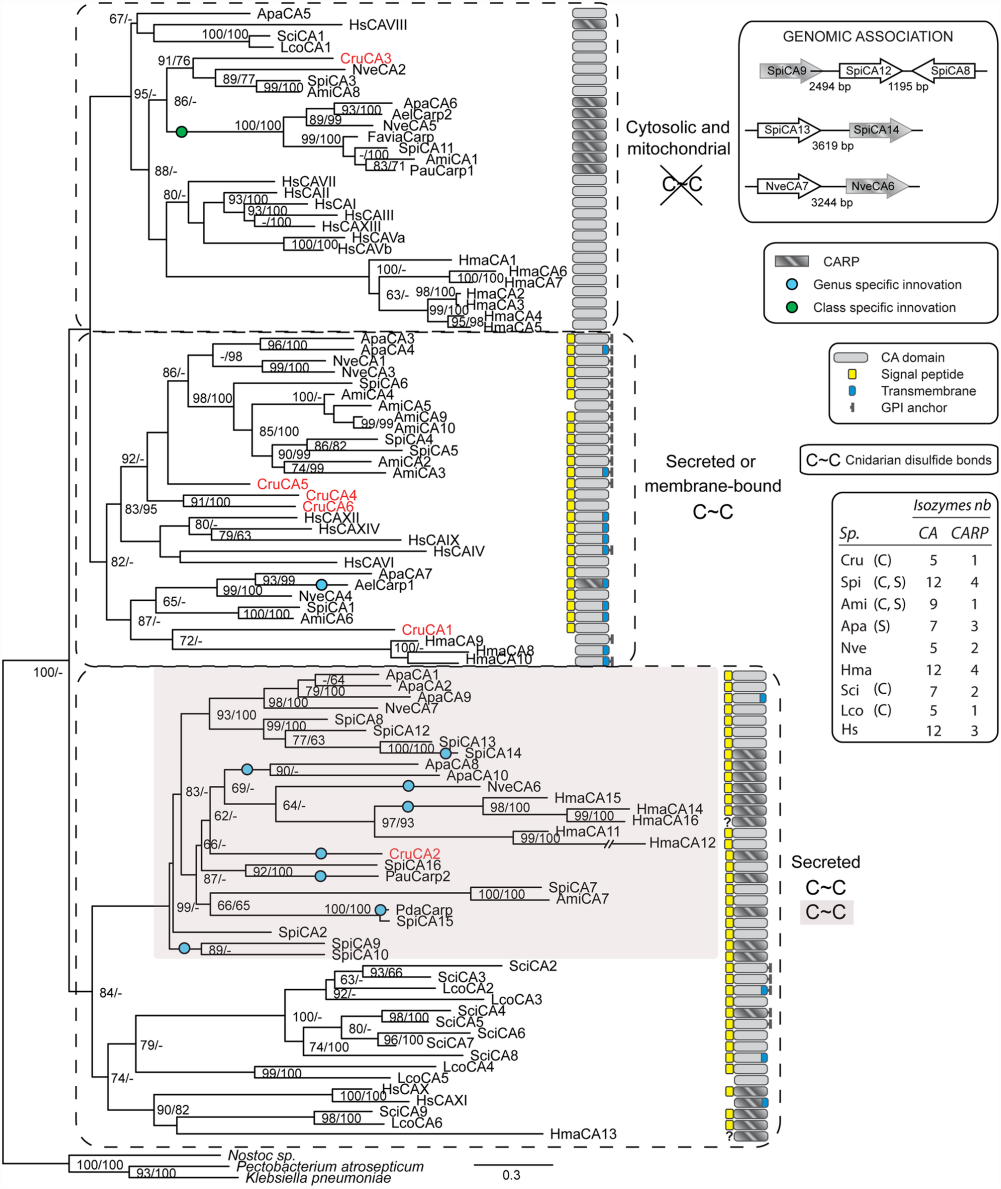
Phylogenetic relationships of 71 cnidarian α-CA protein sequences. α-CAs from human (HsCAI-XIV), two calcareous sponges: *Sycon ciliatum* (SciCA1-9), *Leucosolenia complicata* (LcoCA1-6); and from cnidarians: *Corallium rubrum* (CruCA1-6), *Stylophora pistillata* (SpiCA1-16), *Acropora millepora* (AmiCA1-10), *Nematostella vectensis* (NveCA1-7), *Aiptasia pallida* (ApaCA1-10), and *Hydra magnipapillata* (HmaCA1-15), as well as the CARPs identified in different anthozoan databases, i.e *Anthopleura elegantissima* (AelCARP1-2), *Favia* sp. (FaviaCARP), *Porites australiensis* (PauCARP1-2), *Pocillopora damicornis* (PdaCARP). Sequences were aligned with Clustal Omega and the tree was constructed using PhyML and Bayesian inference methods. The presented topology results from the PhyML method. Node support values indicate PhyML-aLRT values / MrBayes-bootstrap posterior probabilities. Only values above 50% are indicated. The bacterial CA sequences from *Pectobacterium atrosepticum*, *Klebsiella pneumoniae* and *Nostoc sp*. were used as outgroup. Cnidarian α-CAs could be grouped in three main clusters: i) the cytosolic and mitochondrial α-CAs with no disulfide bond, ii) the secreted and membrane bound α-CAs with the canonical disulfide bond, and iii) the secreted α-CAs with two putative disulfide bonds, the canonical and the cnidarian specific disulfide bond. Genes encoding the SpiCA8, 9, 12, the SpiCA13, 14, and the NveCA6, 7 are found closely associated on the same contig. CARP specific innovations and disulfide bonds are indicated for cnidarians. The bottom right inset presents the number of CA and CARP isozymes for each species. *Sp*.: species; *C*: calcifying organism (CaCO_3_ skeletons); *S*: symbiotic organism.

**Fig 5:**
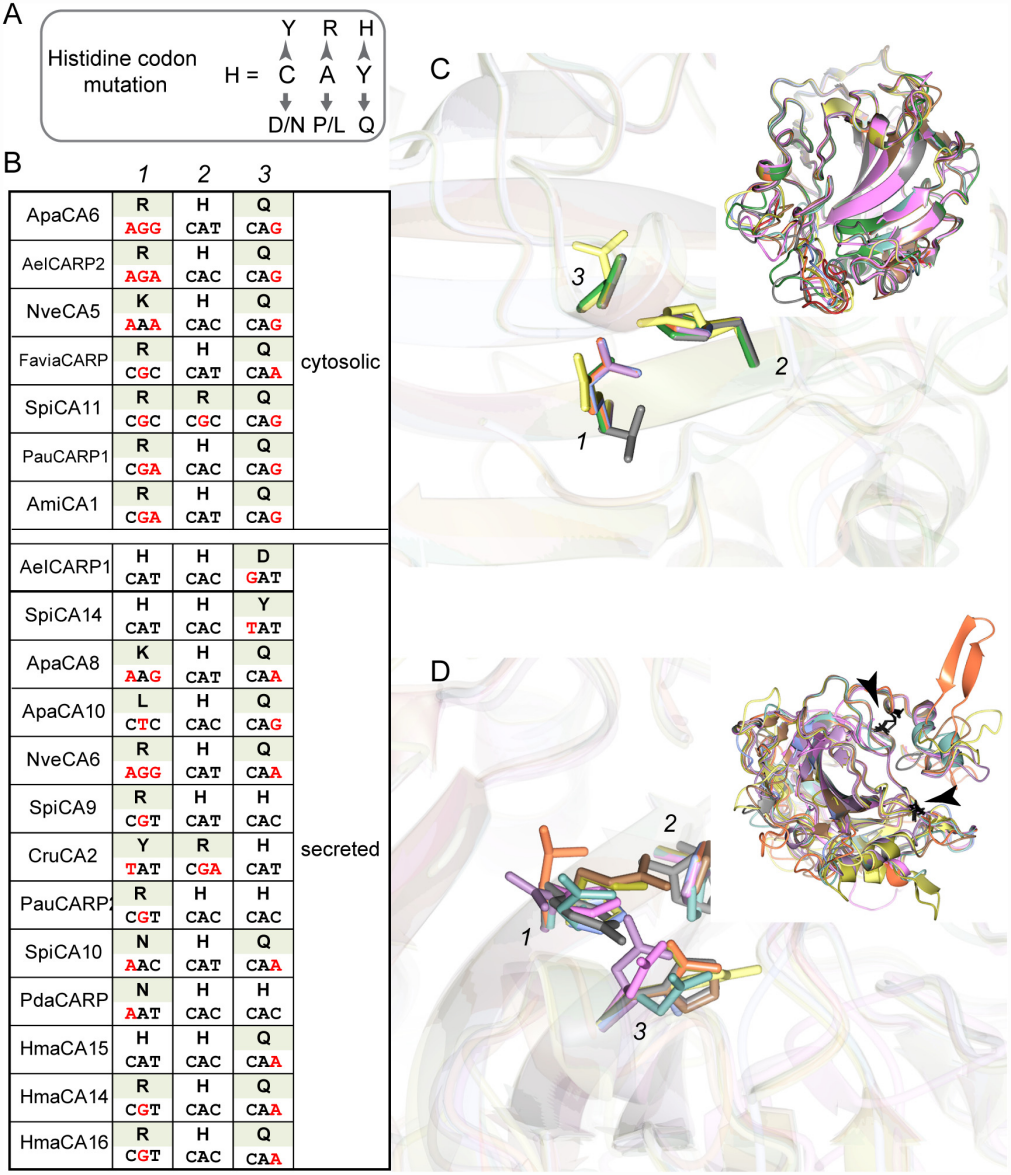
Cnidarian Carbonic Anhydrase Related Proteins (CARPs). (A) The histidine residue is encoded by the codon C.A.C/T. Single mutation resulting from transition (grey arrow head) or transversion (grey small arrow) encodes for the amino acids (one letter code) depicted in the top left panel. (B) Amino acids, and their codons, found in place of the three zinc-binding histidines within the CARP (cytosolic and secreted) identified among cnidarians. (C-D) 3D structure was calculated for each of the full length cnidarian CARP. Pictures are superimposition of the different cytosolic (C) and secreted (D) CARPs models, showing the three residues listed in the left table (B) as well as the overall structure. Black arrowheads indicate the two putative cyst-cyst bridges on the secreted CARPs structure.

Recent development of transcriptomic/genomic data in early-branching metazoans, such as cnidarians, now enables comprehensive analysis within a specific phylum (*e*.*g*. [18,19]). The CruCAs were therefore analyzed within the context of the cnidarian α-CAs identified at the genome scale level. The sequences of the well characterized fifteen human α-CAs as well as the α-CAs from two poriferans (nine from *Sycon ciliatum* and six from *Leucosolenia complicata*) for which complete transcriptome sequences are available, were added to the phylogenetic analysis. For a more complete view of the metazoan α-CAs phylogeny, see references [18,20]. The number of CA isozymes (CARP non-included) was variable between the cnidarian species (like in other species) (Fig 4): five for the calcifying and non-symbiotic octocorallian *Corallium rubrum* (CruCAs) and the non-calcifying and non-symbiotic hexacorallian *Nematostella vectensis* (NveCAs), seven for the non-calcifying and symbiotic hexacorallian *Aiptasia pallida* (ApaCAs), nine and twelve for the calcifying and symbiotic hexacorallians *Acropora millepora* (AmiCAs) and *Stylophora pistillata* (SpiCAs), respectively, and finally twelve isozymes for the non-calcifying and non-symbiotic hydrozoan *Hydra magnipapillata* (HmaCAs). Thus, no apparent correlation between the number of α-CAs and calcification and/or symbiosis could be made. The phylogeny of cnidarian α-CAs (Fig 4) revealed three distinct groups, the intracellular isozymes (cytosolic and mitochondrial) and the extracellular ones which are further separated in two clusters: the secreted α-CAs and the secreted and/or membrane-bound α-CAs. The α-CAs cellular localization, inferred from the protein structure prediction tools (transmembrane domains, GPI anchors and signal peptides), was in direct agreement with their position in the tree (based on the alignment which did not include the localization motifs). Human α-CAs were also present in these three clusters. The poriferan α-CAs were found in only two clusters, the cytosolic and the secreted CAs. However, based on the putative presence of GPI anchor or transmembrane domain in some of the sponges secreted isozymes, these latter should be referred as secreted or membrane-bound α-CAs, instead of secreted α-CAs *senso stricto*. Alignment of the cnidarian α-CAs (S1 Fig) highlighted conserved cysteine residues for putative disulfide bond formation based on their spatial vicinity in the predicted 3D structure (Fig 5, arrowheads): the Cys57~Cys250 (CruCA2 nomenclature) was common to all extracellular isozymes, also conserved in human, sponges and bacteria (S1 Fig); the cytosolic isozymes did not have cysteines at these positions. Another disulfide bridge, corresponding to Cys138~Cys284 was specific to the secreted cluster of α-CAs and appears to be specific to cnidarians.

Notably, the sequence of the *Hydra magnipapillata* CA13 (HmaCA13) was fragmented but based on the partial cysteine signature, it may have been misplaced in the tree and should probably rather be considered as part of the “secreted and membrane-bound isozymes”. We also observed that the α-CAs of actinarians (*N*. *vectensis* and *A*. *pallida*) are closely related, as for the α-CAs of scleractinians (*S*. *pistillata* and *A*. *millepora*). However, one scleractinian specific isozyme conserved between *A*. *millepora* and *S*. *pistillata* (AmiCA7 and SpiCA7) appeared divergent in its primary sequence: although recognized as an α-CA both by Pfam-search and 3D Swiss-Model, and containing the three zinc-binding histidines, it clearly lacked the gatekeeper residues. This isoform also displayed a thirteen amino acids insertion upstream of the proton shuffle histidine, suggesting an unusual catalytic activity for this secreted isozyme, if any. Finally, the α-CA isozymes of *C*. *rubrum* appeared to branch at intermediate positions within the different clades of the tree, supporting the evolutionary position of octocorals.

### Carbonic Anhydrase Related Proteins

CARPs were found in every species investigated and, similar to the α-CAs, their number was variable across sampled cnidarian species. We noticed that the cnidarian CARPs were clustered inside the group of intracellular isozymes but appeared polyphyletic in the case of the secreted CARPs. This arrangement was confirmed by different alignment methods (S2 Fig). We then included in our phylogenetic analysis different CARPs present in the anthozoan subdivision of the NCBI EST/TSA databases, *i*.*e*. *Anthopleura elegantissima* (symbiotic; AelCARP1-2), *Favia* sp. (calcifying and symbiotic; FaviaCARP), *Porites australiensis* (calcifying and symbiotic; PauCARP1-2), *Pocillopora damicornis* (calcifying and symbiotic; PdaCARP). Two noteworthy aspects of the cnidarian CARPs evolution became evident: 1) cytosolic CARPs seem to be restricted to Hexacorallia and monophyletic; no cytosolic CARPs were obtained from *C*. *rubrum* or *H*. *magnipapillata*; 2) extracellular CARPs had been reinvented multiple times during the course of cnidarian evolution as they were scattered along the cluster of secreted cnidarian α-CAs, inferring class or genus specific gene innovations. The same holds true for the *S*. *ciliatum* SciCA4. Furthermore, the CARPs SpiCA14, SpiCA9, and Nve6 appeared encoded in tandem array with the α-CAs SpiCA13, SpiCA12 and Nve7, respectively. As evidenced both by phylogenetic tree (Fig 4) and protein distance matrices (S2 and S3 Tables), SpiCA13 and SpiCA14, at least, resulted from a recent duplication. In the same line of evidences, the coral CARPs from *P*. *damicornis* (PdaCARP) and *P*. *australiensis* (PauCARP2) likely represent evolution of the *S*. *pistillata* α-CAs SpiCA15 and SpiCA16, respectively. Finally, the *A*. *elegantissima* CARP1 (AelCARP1) turned out to be a membrane-bound CARP, unique in its cluster.

Remarkably, apart from the three zinc-binding histidine residues that were mutated in one or two positions, we could not find typical signature for the CARP sequences, enabling to differentiate them from the α-CAs. However, we found that the His-His-His (H-H-H) zinc-binding signature was often converted toward Arg-His-Gln (R-H-Q), arguing against random genomic mutations in the histidine codon (Fig 5*A* and 5*B*). This was especially true for the cytosolic CARPs. Although those are expected to have arisen from a common ancestor, examination of the Arg-His-Gln codon sequences shows accumulation of both transitions and transversions, maintaining this specific CARP triplet signature. Moreover, 3D modeling of the cytosolic and the secreted CARPs showed that the overall structure of the cytosolic CARPs was globally conserved with a fairly well conserved “acatalytic site” (Fig 5*C* and S1 and S2 Movies), as opposed to the secreted forms for which conservation was less obvious (Fig 5*D* and S3 and S4 Movies). Thus, our study provides support for a positive selection of the cytosolic CARPs in Hexacorallia, and for a recurrent reinvention of the secreted CARPs with apparently little structural constraints.

## Discussion

The genome and transcriptome of the adult Mediterranean red coral, *C*. *rubrum*, are currently at an assembly stage permitting unambiguous data mining of specific genes (PG, DZ, DA, ST, personal communication). Among the class of Anthozoa (phylum Cnidaria), *C*. *rubrum* is part of the Octocorallia, the sister group to Hexacorallia which includes the calcifying reef-building corals and the non-calcifying sea anemones. *C*. *rubrum* builds two calcareous biominerals, making it a model of choice for the investigation of the calcification process and its evolution within cnidarian. α-CAs are central to biomineralization in metazoans and are the subject of the present study focusing on cnidarians.

### Identification and tissular expression of α-CAs in *C*. *rubrum*

**Fig 6:**
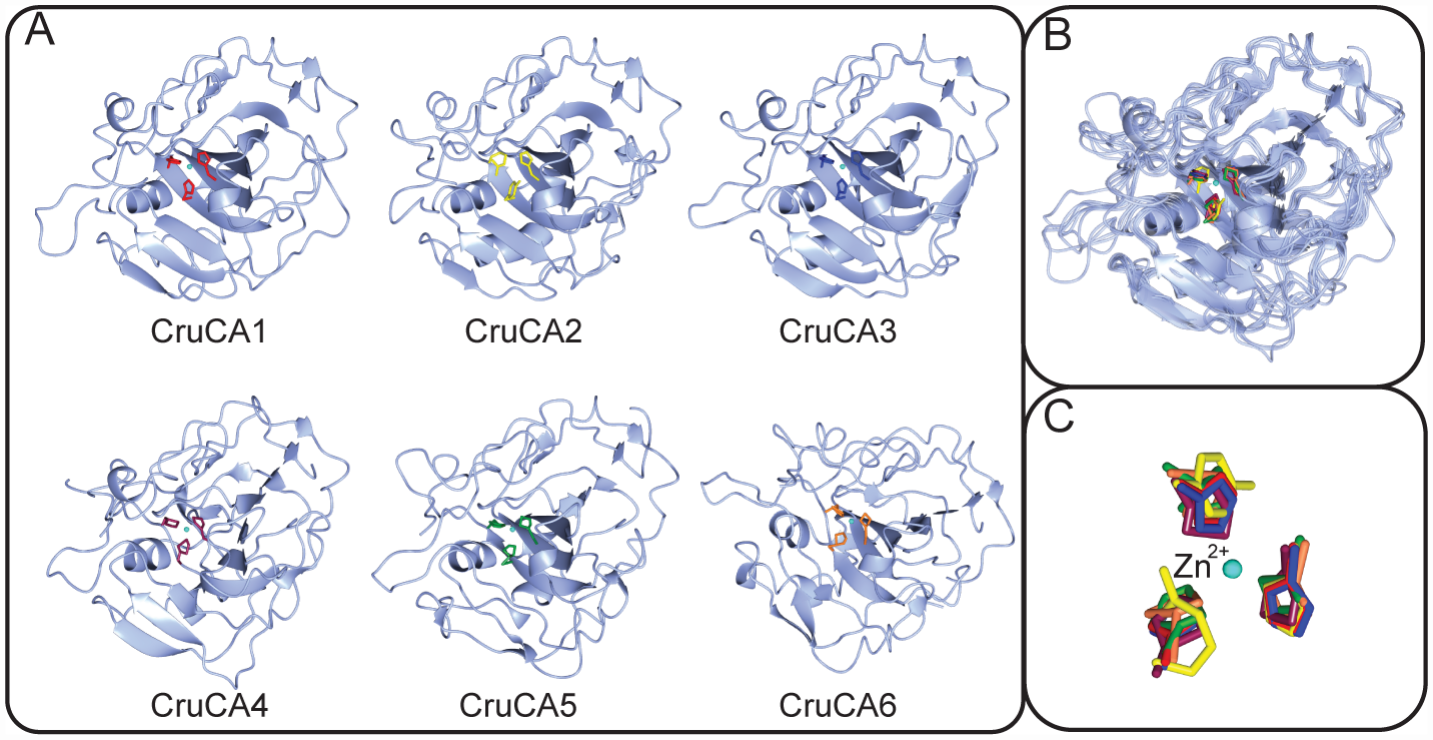
Predicted 3D structure of CruCA isozymes. (A) Ribbon diagram of each CruCA 3D structure modeling. The three histidines coordinating the zinc ion at the active site are shown in bright colors. The “acatalytic” isoform, CruCA2, displays histidine, arginine and tyrosine residues in yellow. (B) Superimposition of the six CruCAs showing their high structural homology. (C) Superimposition of the three zinc-binding histidines of each CruCA, and histidine, arginine and tyrosine for CruCA2 in yellow.

Data mining of the genome and transcriptome of *C*. *rubrum* allowed identification of a total of six genes encoding α-CA isoforms. This low number of isoforms is an asset to study their respective role in the animal. The genes and their corresponding protein sequences were named CruCA1 to 6. All isozymes could be folded with a core domain composed of parallel β-sheets surrounded by several short α-helices and β-strands, in a typical 3D α-CA structure (Fig 6). Interestingly, the proton shuttle residue is substituted in CruCA1-3-4-5; α-CAs which lack this histidine are characterized by much lower hydrase efficiency [62]. CruCA3 is the only cytosolic α-CA and ubiquitously expressed in the two compartments that we considered, *i*.*e*. the non-calcifying polyps and the calcifying tissues. CruCA5 is the most abundantly expressed α-CA, predominantly expressed in the polyps. It is a membrane-bound α-CA and displays a forty two amino acids linear structure, containing multiple phosphorylation and glycosylation sites, between the predicted anchoring residue and the globular CA domain. CruCA1 and 6 are secreted α-CAs preferentially expressed in the polyps, with expression of CruCA6 being very low. CruCA2 lacks the two functionally important histidines and is thus considered as a CARP, specific to the non-calcifying tissues. Interestingly, CruCA4, which appears to share a common ancestor with CruCA6, is secreted and is the only isozyme specifically expressed in the calcifying fraction. In light of these results, CruCA4 represents the top candidate for an α-CA involved in calcification of biominerals of *C*. *rubrum*.

Although we cannot exclude that diel cycle may influence CruCAs expression patterns, previous studies on *C*. *rubrum* showed constant calcification rates over several days [49,63], indicating that red coral calcification-related processes may not be associated to circadian rhythms. In addition, this species, contrary to scleractinian corals, does not possess any photosynthetic endosymbiont and lives in dimly lit habitats, such as caves and smaller cavities, thereby minimizing direct light influence. On the other hand, CruCAs may be regulated during development (e.g. CruCA6) as studies in *Acropora millepora* identified two CAs specifically upregulated at the planula and post-settlement stages [64]. Developmental expression studies on *C*. *rubrum* should be carried out to complement the spatio-temporal picture of CAs expression.

### Phylogenetic analysis of cnidarian α-CAs

One significant difficulty to construct a robust phylogenetic tree of metazoan α-CAs is the lack of full genome and transcriptome permitting the identification of full CA sequences in representative species [18,20]. In the present study, we choose to construct the phylogenetic tree of cnidarian α-CAs using both whole genome/transcriptome from published or soon to be published cnidarian species. The speciation between the cytosolic, secreted and membrane-bound, and the secreted CAs was demonstrated using the three following criteria: 1) the distribution of the conserved carbonic anhydrase domain sequences along the phylogenetic tree, 2) the presence/absence of a signal peptide, a trans-membrane domain, and/or a GPI anchoring, outside the CA domain, and 3) the presence of 0, 1 or 2 putative disulfide bridges in the cytosolic, secreted and membrane-bound, and the secreted isozymes, respectively. Interestingly, the second disulfide bridge, conserved among the secreted cluster, is to our knowledge, specific to cnidarians. This raises functional questions in regard to the secreted isozymes phylogenetically associated with the membrane-bound ones that do not display this second disulfide bridge (namely CruCA1, 4, 6, ApaCA7, NveCA4). Alternatively, the two clusters of secreted isozymes could reflect distinct localizations with different pH constraints, such as the acidic intracellular vesicles (endosomes, lysosomes…) and the extracellular medium.

### Comparative analysis of the cnidarian Carbonic Anhydrase Related Proteins (CARPs)

One of the surprising outcomes of our study was the identification of cytosolic and secreted CARPs clearly reinforcing the view that CARPs represent evolutionary conserved proteins (or at least conserved function) identified in poriferans [19], hexacorallians, one octocorallian (this study), placozoans and bilaterians [18,65]. However, they do not appear to have evolved from a common ancestor since in cnidarians, both the cytosolic and the secreted isozymes are clade specific. More surprising was the evidence that the secreted CARPs had been reinvented multiple times during the course of cnidarian evolution, suggesting a functional constraint. Besides, we also showed a trend toward R-H-Q substituting the canonical H-H-H zinc binding triplet, especially true for the cytosolic isozymes. This R-H-Q triplet is again found in CARPs from various metazoan species including vertebrates (CARP X, [65]), sea urchins (UniProt #W4XL56), oysters (UniProt #E5RQ31 [66]) and calcareous sponges [19]. A more permissive triplet sequence R/H/Y-H/R-Q/H would account for a majority of the CARPs found in the metazoan sequence databases (not shown). This, combined with the facts that 1) reversing the substituted triplets of the human CARPs to H-H-H restores their catalytic activity [67], and 2) CARP knock down in zebrafish results in abnormal brain development and function [68,69], highlight their functional requirement. Interestingly, cnidarian possess no equivalent tissues to the liver or lungs but possess a simple nervous system [70], where vertebrates’ CARPs are mostly expressed [17,65]. On the other hand, we showed the secreted forms of cnidarian CARPs have poor 3D structure conservation, especially in regards to the active site conformation. Thus, in light of our results, two functional categories could be conceived, one with an evolutionary conserved cellular/physiological function as depicted by the conserved R-H-Q triplet (or near variants) and another one which may be cnidarian specific, as depicted by the pattern of recurrent invention (convergent neofunctionalization?) for an extracellular function. Of thoughts, in our cnidarian species sampling, every secreted catalytic α-CA had a secreted CARP counterpart, which could represent an acatalytic competitor for functional negative regulation purposes.

### Carbonic anhydrases and calcification in corals

In the present study, since we could determine the number of α-CAs in corals, we asked whether a higher number of α-CAs may be linked to a higher calcifying activity. We thus compared the slow calcifying species *C*. *rubrum* (calcification rate of 21 nmol of Ca^2+^.d^-1^.mg^-1^ protein [49]), with the reef-building fast-growing species *S*. *pistillata* (calcification rate around 3000 nmol of Ca^2+^.d^-1^.mg^-1^ protein [71]) and found that the number of catalytically active α-CAs is five in *C*. *rubrum* and twelve in *S*. *pistillata*. This result suggests that a higher number of α-CAs is linked to a higher calcifying activity. However when considering a non-calcifying species (*H*. *magnipapillata*), we found the same number of α-CAs as in *S*. *pistillata* (12 CAs) which does not support the link between calcification and number of α-CAs. When considering the role of α-CAs in coral calcification, the reef-building coral *S*. *pistillata* is certainly the calcifying cnidarian for which the set of data is the most abundant as data result from both physiological and molecular studies (for reviews see [34]). In this species it has been proposed that two isozymes are involved in calcification: one secreted isozyme named STPCA [23] and one intracellular isozyme named STPCA2 [50], which respectively correspond to SpiCA1 and SpiCA2 in this study’s nomenclature. Both were immunolocalized in tissues but also inside the skeleton for SpiCA2 [31], suggesting a structural role in calcification. In *A*. *millepora*, the membrane bound AmiCA4 and the cytosolic AmiCA8 were suggested to play a role in the onset of the post-settlement calcification [64]. Interestingly none of the α-CAs shown to be involved in reef-building corals calcification (*S*. *pistillata* and *A*. *millepora*) can be evolutionary linked to CruCA4, the candidate that we identified as specially expressed in the calcifying tissues of *C*. *rubrum*. The evolution of α-CAs in metazoans was driven by frequent gene diversification and gene loss events [19]. This highly dynamic evolution makes α-CAs eligible for repeated independent recruitment in novel physiological functions, such as biomineralization, in different metazoan lineages [19]. To go further, our results support an independent recruitment of α-CAs for the biomineralization process within anthozoans, which likely occurred after the Octocorallia- Hexacorallia speciation.

### Potential role of α-CAs in biomineralization of *C*. *rubrum*

Based on a pharmacological approach, it has been shown that CAs are involved in the formation of both sclerites and skeleton in *C*. *rubrum* [49]. This study also suggested that at least one CA involved in calcification should be intracellular. This intracellular CA would catalyze the hydration of metabolic CO_2_ (provided by respiration) into HCO_3_^-^, which is then transported to the site of calcification. The only intracellular CruCA identified in this study, CruCA3, does not show any differential expression between tissues enriched in calcifying cells and polyps. However this does not preclude a role in biomineralization for CruCA3. Indeed, change in intracellular CA expression/activity will affect cell homeostasis including modification of the acid-base equilibrium which will undoubtedly affect calcification. Also, this intracellular CA may be associated with bicarbonate transport as part of a supramolecular transport complex (metabolon [72–74]), and will provide directional transport, *e*.*g*. towards the calcification site, if preferentially localized at one side of the cell. Subcellular localization and molecular association would be necessary to provide further details on the role of this CruCA3 in the different tissue layers of *C*. *rubrum*.

In the present study, as shown both by qPCR and western blot, CruCA4 is the only secreted isozyme preferentially expressed in tissues enriched in calcifying cells. This result suggests that CruCA4 is involved in calcification even if the expression of this isozyme is low compared to other isozymes. This low level of expression can be due to the fact that *C*. *rubrum* is a slow calcifying species [49]. Another possibility lies in the fact that the volume of calcifying cells would be low compared to that of the other non-calcifying cells, thus introducing a bias. Whatever the reason of its low expression, this does not preclude that this isozyme can be extremely efficient, meaning with a high catalytic activity. Further biochemical studies (proteomic, enzymatic, immunochemistry) are necessary to elucidate the exact role of CruCA4 in calcification.

In summary, by combining data mining of the genome/transcriptome of *C*. *rubrum* with quantitative gene expression, we have characterized the complete set of α-CAs showing that, among the six isozymes identified, one is a cytosolic isozyme (CruCA3), one is a CARP isozyme (CruCA2), one is a calcifying specific secreted isozyme (CruCA4) and one is a membrane-bound isozyme (CruCA5). Besides, phylogenetic analysis of our updated data shows an independent recruitment of α-CAs for calcification in cnidarians. Finally, the study of cnidarian CARPs highlighted evolutionary conserved motifs.

## Supporting Information

S1 Appendix Clustal Omega alignment of α-CAs in Nexus format. Alignment of α-CAs used to produce the Bayesian tree. The two disulfide bridges are indicated as comments, uppercase “C” for the bridge common to all extracellular α-CAs, and lowercase “c” for the one conserved among the secreted α-CAs cluster and specific to cnidarians.
(NEX)

S1 Fig Clustal Omega alignment of α-CAs. The two disulfide bonds are highlighted: (1) common to all extracellular α-CAs (highlighted in yellow) and (2) conserved among the secreted α-CAs cluster and specific to cnidarians (highlighted in green).
(PDF)

S2 Fig Phylogenetic trees of α-CAs aligned with Muscle or MAFFT and built with PhyML. Cytosolic and secreted CARPs are written in green and blue, respectively. Branches that are non-congruent with the tree constructed from Clustal Omega (S1 Fig and Fig 4) are colored in red. Despite the little incongruence between the trees produced by the different alignment methods, the secreted CARPs always appear polyphyletic within the secreted α-CAs cluster.
(TIF)

S1 Movie Superimposition of all modeled cytosolic cnidarian CARPs. The movie shows that the global structure is well conserved.
(MPG)

S2 Movie Superimposition of the catalytic site of modeled cytosolic cnidarian CARPs. A close-up view of the residues present in place of the three zinc-binding histidines, the threonine gate keeper residue, and the proton shuttle residue of catalytic α-CAs.
(MPG)

S3 Movie Superimposition of all modeled secreted cnidarian CARPs. The movie shows that, contrary to the cytosolic CARPs, their global structure is more divergent. However, two Cyst-Cyst bridges can be envisaged (black side chains cysteine residues).
(MPG)

S4 Movie Superimposition of the catalytic site of modeled secreted cnidarian CARPs. A close-up view of residues present in place of the three zinc-binding histidines of catalytic α-CAs showing their spatial arrangement.
(MPG)

S1 Table Accession numbers of α-CA sequences, and templates used for 3D structure modelling of CruCAs and full length anthozoans CARPs. Nucleotide sequences of newly identified α-CAs for *C*. *rubrum*, *S*. *pistillata*, and *A*. *pallida* are reported here.
(XLSX)

S2 Table Sequence identity matrix of cnidarian α-CAs. Sequence pair distances of cnidarian α-CAs generated from the alignment described in S1 Fig and generated with the program Bioedit. The values are percentage amino acid identities of a given sequence pair. For CARPs, the best score per column is highlighted in yellow, and the two best scores are in bold.
(XLSX)

S3 Table Estimates of evolutionary divergence of cnidarian α-CAs. The number of amino acid substitutions per site between sequences is shown. Analyses were conducted using the Poisson correction model. The analysis involved 72 amino acid sequences. All positions with less than 95% site coverage were eliminated. That is, fewer than 5% alignment gaps, missing data, and ambiguous bases were allowed at any position. There were a total of 146 positions in the final dataset. Evolutionary analyses were conducted in Mega (V6.0). For CARPs, the best score per column is highlighted in yellow, and the two best scores are in bold.
(XLSX)
