# Supplementary figures and images for: Carbonic Anhydrases in Cnidarians: Novel Perspectives from the Octocorallian Corallium rubrum
Source: PLoS One. 2016 Aug 11;11(8):e0160368. doi: 10.1371/journal.pone.0160368 (PMC4981384; doi:10.1371/journal.pone.0160368)

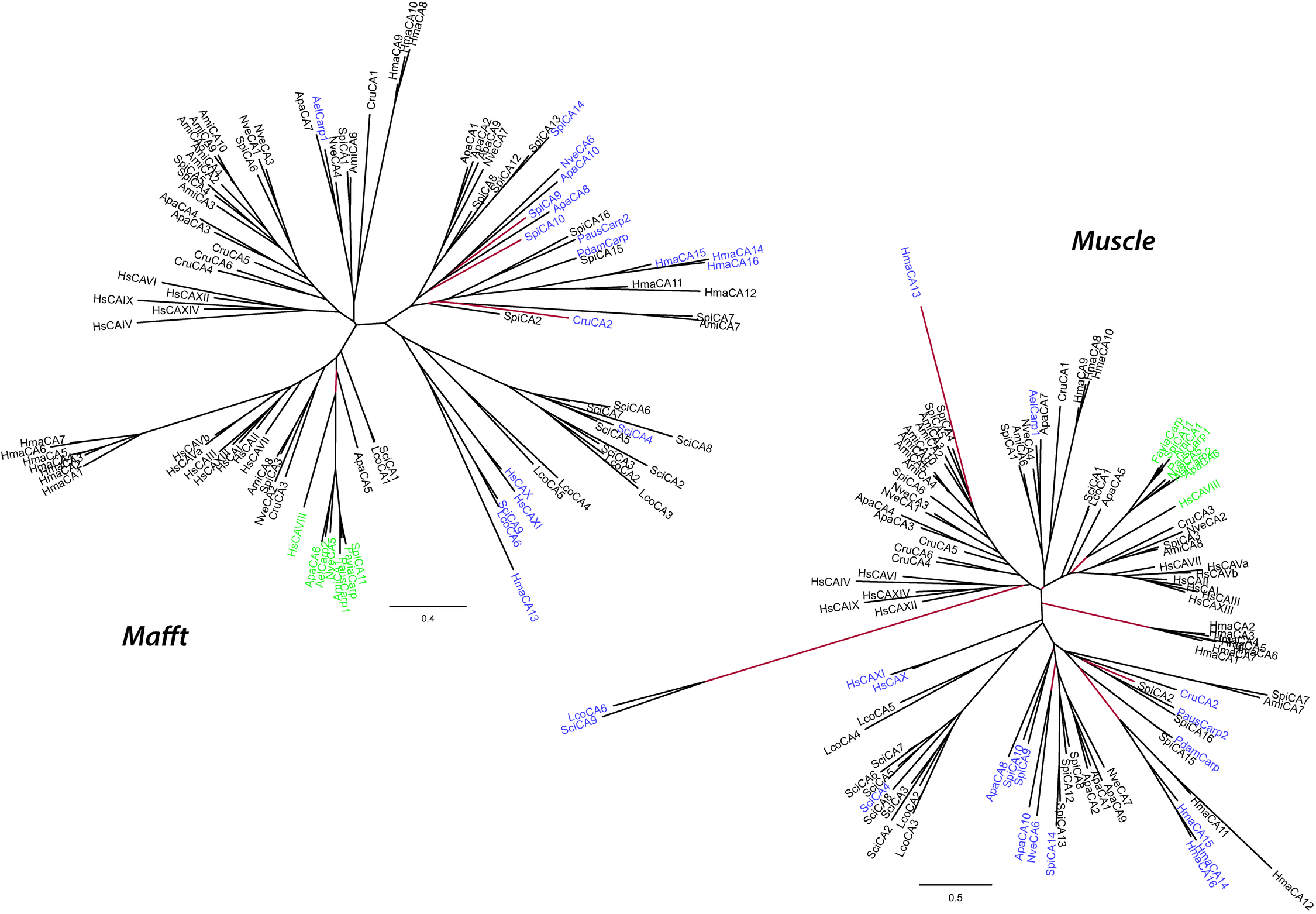

Supplement: S2 Fig — Cytosolic and secreted CARPs are written in green and blue, respectively. Branches that are non-congruent with the tree constructed from Clustal Omega (S1 Fig and Fig 4) are colored in red. Despite the little incongruence between the trees produced by the different alignment methods, the secreted CARPs always appear polyphyletic within the secreted α-CAs cluster. (TIF) [file pone.0160368.s003.tif]
